# Creutzfeldt-Jakob disease with Alzheimer pathology, presenting with status epilepticus following repeated partial seizures: a case report and literature review

**DOI:** 10.1186/s12883-018-1055-y

**Published:** 2018-04-25

**Authors:** Keita Miyake, Takashi Hara, Etsuko Oshima, Kiyohiro Kawada, Hideki Ishizu, Yuko Yamauchi, Katsuya Satoh, Tetsuyuki Kitamoto, Shintaro Takenoshita, Seishi Terada, Norihito Yamada

**Affiliations:** 1 Department of Psychiatry, Okayama University Graduate School of Medicine, Dentistry, and Pharmaceutical Sciences, 2-5-1 Shikata-cho, Kita-ku, Okayama, 700-8558 Japan; 2 Department of Psychiatry, Hayashi Hospital, Okayama, Japan; 3 Department of Psychiatry, Zikei Hospital, Okayama, Japan; 4 Department of Locomotive Rehabilitation Sciences, Nagasaki University Graduate School of Medicine, Nagasaki, Japan; 5 Department of Neurological Science, Tohoku University Graduate School of Medicine, Sendai, Japan

**Keywords:** Alzheimer disease, Creutzfeldt-Jakob disease, Epilepsy, Seizure, Status epilepticus

## Abstract

**Background:**

Creutzfeldt-Jakob disease (CJD) is a fatal neurodegenerative disease. Common first symptoms are dementia, cerebellar ataxia, visual disturbance, and psychiatric symptoms. Seizure as the first symptom of CJD is a very rare finding.

**Case presentation:**

We experienced an elderly woman who presented initially with status epilepticus following repeated partial seizures in the course of Alzheimer disease (AD) dementia. Anti-convulsive therapy had no effect. Autopsy revealed definite CJD with AD pathology.

**Coclusions:**

This is the first reported CJD case presenting with status epilepticus in the course of AD dementia.

## Background

Creutzfeldt-Jakob disease (CJD) is a fatal neurodegenerative disease affecting humans and a wide variety of animals. Most cases are sporadic with an unknown etiology. The incidence of CJD is approximately one case per million person per year [1]. The typical presentations include rapidly progressive cognitive decline, behavioral changes, cerebellar dysfunction, and visual disturbances [1]. Epilepsy is an uncommon feature in patients with CJD [2]. Here, we report an elderly woman with CJD who presented initially with status epilepticus (SE) following repeated partial seizures during the course of Alzheimer disease dementia (ADD).

## Case presentation

**Fig. 1:**
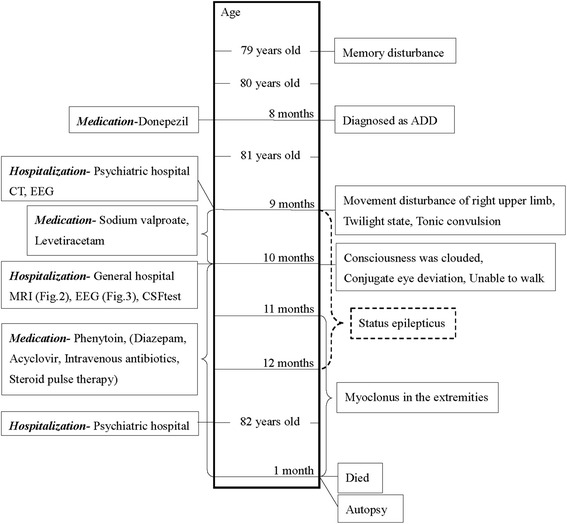
Medical history of the patient displayed on the timeline

The patient was a Japanese woman who was 82 years and 1 month old at the time of death. She had no family history of dementia, and had not received dura mater transplantation. Medical history of the patient are summarized on a timeline (Fig. 1).

She developed memory disturbance at the age of 79 and became unable to pay invoices. She sometimes complained of money being stolen, and had a check-up at memory clinic. The scores of the Mini Mental State Examination and Hasegawa Dementia Scale Revised were 19/30 and 14/30, respectively. She was diagnosed with ADD, and started to take donepezil orally at the age of 80 years and 8 months. For 13 months after the start of donepezil treatment, no remarkable changes were observed in her cognitive and motor function.

At the age of 81 years and 9 months, she became unable to move her right hand well, and her right arm sometimes elevated involuntarily. She declined into a twilight state gradually over two weeks. She spoke ambiguously and did not make eye contact. Head CT showed no remarkable change. She was hospitalized in a ward for patients with dementia in a psychiatric hospital. She had no fever. Electroencephalogram (EEG) showed spikes in the left parietal region, and slow wave bursts in the bilateral frontal areas. Tonic convulsion in the right upper extremity occurred several times a day. Sodium valproate (400 mg/day) and levetiracetam (1000 mg/day) were started. Her consciousness level fluctuated and was not clear all day, and she gradually became unable to walk. One week after admission, at the age of 81 years and 10 months, she was transferred to a general hospital.

Consciousness was clouded, and she was bed-ridden. Body temperature was 38.1 degrees. The Glasgow coma scale score was 7 (eye opening 3, verbal response 1, motor response 3), and the Japan coma scale score was 30. The right upper extremity was held in a flexed posture and did not move. The left upper extremity and bilateral lower extremities moved voluntarily. Deep tendon reflex was within normal range, and Babinski sign was negative. Conjugate eye deviation to the right and tonic convulsion in the right upper limb for a few minutes was observed about ten times a day.

**Fig. 2:**
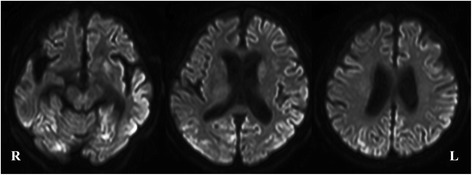
Head MRI (axial, diffusion-weighted image) of the patient. Diffusion-weighted brain images showed high intensity lesions, separately in the cortex of the left temporo-parietal lobe, right parietal lobe, left frontal lobe, left insula, and left basal ganglia

Cerebrospinal fluid (CSF) examination revealed a normal initial pressure (180 mmHg), normal cell count (3/3), slightly elevated protein level (65 mg/dl), and normal sugar level (45 mg/dl). The IgG index was low (0.375), and the neuron-specific enolase concentration in CSF was elevated (44.24 ng/ml). Anti-thyroid peroxidase and anti-thyroglobulin antibodies were negative. Head diffusion magnetic resonance imaging (MRI) showed ribbon-like high intensity areas, separately in the cortex of the left temporo-parietal lobe, right parietal lobe, left frontal lobe, left insula, and left basal ganglia (Fig. 2). T1- and T2-weighted images of the head MRI showed no remarkable lesions except for the severe bilateral hippocampal atrophy (VSRAD advance: severity of hippocampal atrophy, 2.62) [3]. EEG showed diffuse periodic slow waves, similar to periodic lateralized epileptiform discharges, in the left hemisphere dominantly.

**Fig. 3:**
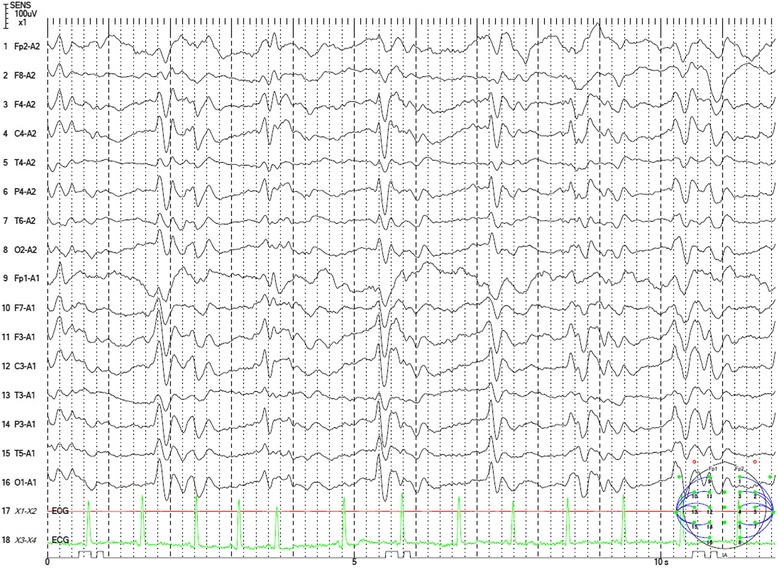
Electroencephalogram of the patient. Electroencephalogram showed periodic synchronous discharge

SE was strongly suspected, and intensive anti-convulsive therapy with intravenous phenytoin and diazepam suppository was started. Additionally, intravenous antibiotics and acyclovir were used for a week. However, no remarkable improvement was observed in the state of the patient. Tonic convulsion in the right upper limb with a decrease of oxygen saturation for a few minutes several times a day was observed throughout the course. Steroid pulse therapy for three days was performed twice, but had no effect. Gradually tonic convulsion decreased, and myoclonus appeared in the lower extremities. The EEG evolved into a more generalized periodic synchronous discharge (PSD) (Fig. 3).

One month later, she was returned to a psychiatric hospital at the age of 81 years and 11 months. She developed akinetic mutism at the age of 82 years, and she died at the age of 82 years and one month.

An autopsy was done three hours after death, and the brain weight was 980 g. Macroscopically, diffuse cortical atrophy was found. No atrophy was observed in the cerebellum or the white matter of the cerebrum. Microscopically, early lesions with typical spongiform changes in the cerebral cortex were found diffusely, and slight vacuolations were observed in the frontal and occipital lobes. An immunohistochemical study for prion protein showed synaptic deposition diffusely in the cerebrum, and a slightly perivacuolar pattern was observed in the frontal and occipital lobes. In the cerebellum, slight spongiform changes were found in the molecular layer of the cerebellum, and synaptic depositions of prion protein were observed. AD-related neurofibrillary pathology of this case was Stage IV-V, and the amyloid phase was Phase 5 [4, 5].

Genetic testing showed no mutations and a 129Met/Met, 219Glu/Glu pattern in the prion protein gene. Western blot showed an MM1 pattern. In the CSF, 14–3-3 protein was negative, and RT-QuIC of prion protein was positive. The genotype of apolipoprotein E was e3/e4.

## Discussion and conclusion

The coexistence of AD pathology and prion pathology in the same case is uncommon [6]. Brown et al. presented a synopsis of a National Institute of Health series of 189 CJD cases autopsied during the past 30 years, and only four of them were found to exhibit evidence of AD pathology [7]. Tsuchiya et al. stated that there were two forms of coexistence of CJD and AD [8]. The first type is AD cases developing CJD in the late stage of AD, and the second is CJD having AD pathology without clinical features of AD [8]. Our case corresponds to the first form.

AD is associated with a high risk for developing epileptic seizures [9]. AD and other neurodegenerative conditions represent the presumed etiology for 10% of new onset epilepsy in patients older than 65 [10]. Therefore, in the beginning, we suspected epileptic seizures due to AD in this case. Because anti-epilepticus treatment had no effect and a gradually worsening consciousness level was observed, we reconsidered the cause of the seizures and suspected other conditions such as encephalitis or encephalopathy.

Although about 70% of aged patients with SE were without a previous diagnosis of epilepsy, diffusion-weighted hyperintensities associated with SE were observed in 28% of patients (26 of 93 patients) [11]. A ribbon-like high intensity area in the cerebral cortex on diffusion-weighted images might be induced by SE [11]. Therefore, we broadly examined the cause of SE and performed intensive anti-convulsive therapy. However, there was no obvious effect.

As stated above, Yoshimura et al. found abnormal hyperintensities on diffusion-weighted images in 28% of the patients with SE [11]. In 25 of the 26 patients, diffusion-weighted hyperintensities were unilateral. Only one case had bilateral thalamic lesions. In our case, diffusion-weighted hyperintensities were found separately in left temporo-parietal, right parietal, left frontal lobes, left insula, and left basal ganglia. If the case showed bilateral diffusion-weighted hyperintensities, we had better consider a cause other than simple SE.

In two large surveillance reports contaning 674 or 492 sporadic CJD cases, there was no case with an epileptic seizure as the first symptom of CJD [12, 13]. Only two of 492 sporadic CJD cases showed myoclonus as the first symptom [13]. In 264 patients initially suspected of having prion disease but finally judged not to, 18 cases were diagnosed as epilepsy and two were diagnosed as AD and epilepsy [14]. It was difficult for us to diagnose CJD in the early stage because we did not recognize the presence of rapidly progressive dementia due to SE. After the appearance of the myoclonic jerk in the lower extremities and PSD on EEG, we diagnosed her as having probable CJD.

Seven patients with CJD who presented with partial seizures as an initial symptom have been reported (Table 1). In most cases, anti-convulsive therapy had no effect. Our patient was much older than the other six cases, and was the only case who suffered from early partial seizures due to CJD during the course of ADD. Like the previous reports, no pathological differences were found between the epileptogenic region and other cortices [15, 17, 18]. Although complex partial seizures and SE are rare in CJD, we should consider CJD in the differential diagnosis in aged patients with SE.

**Table 1:** CJD patients who presented with partial seizures as an initial symptom

Case	Age	Sex	Seizures	Automatism	Effect^a^	Autopsy^b^	Type^c^
Rees et al., 1999 [15]	58	F	CPS	(−)	(−)	autopsy	nm
Rees et al., 1999 [15]	68	M	CPS	(−)	(−)	(−)	(−)
Lee et al., 2000 [16]	42	M	CPS, EPC	jerky movement of right arm	(−)	biopsy	nm
Parry et al., 2001 [17]	67	F	EPC	flailing of the left upper limb	(+)	autopsy	nm
Neufeld et al., 2003 [18]	62	M	SPC, SGS	(−)	(−)	autopsy	E200K
Ogawa et al., 2011 [19]	61	F	CPS	hand and oral automatism	(−)	(−)	(−)
Miyake et al., 2017	82	F	CPS	elevation of right arm	(−)	autopsy	MM1

Effect^a^, effect of anti-epileptics; Autopsy^b^, autopsy or biopsy; Type^c^, type of CJD

*CPS* complex partial seizure, *EPC* epilepsia partialis continua, *SPC* simple partial seizure, *SGS* secondary generalized seizure, *nm* not mentioned

## Abbreviations

AD: Alzheimer disease
ADD: Alzheimer disease dementia
CJD: Creutzfeldt-Jakob disease
CSF: Cerebrospinal fluid
EEG: Electroencephalogram
MRI: Magnetic resonance imaging
PSD: Periodic synchronous discharge
SE: Status epilepticus
